# Development of Voriconazole Proliposome Based Dry Powder for Inhalation: A Design of Experiment Approach

**DOI:** 10.3390/pharmaceutics17050622

**Published:** 2025-05-08

**Authors:** Sanjeevani Deshkar, Alisha Vas, Roshani Pagar, Prabhanjan Giram, Asha Thomas, Vaishali Undale

**Affiliations:** 1 Department of Pharmaceutics, Dr. D. Y. Patil Institute of Pharmaceutical Sciences and Research, Pune 411018, Maharashtra, Indiaroshani0428@gmail.com (R.P.); 2 Department of Pharmaceutical Sciences, The State University of New York, Buffalo, NY 14214, USA; 3 Department of Pharmaceutical Chemistry, Dr. D. Y. Patil Institute of Pharmaceutical Sciences and Research, Pune 411018, Maharashtra, India; asha.thomas@dypvp.edu.in; 4 Department of Pharmacology, Dr. D. Y. Patil Institute of Pharmaceutical Sciences and Research, Pune 411018, Maharashtra, India; vaishali.undale@dypvp.edu.in

**Keywords:** proliposome, spray drying, design of experiment, in vivo lung retention study, voriconazole, dry powders for inhalation, pulmonary drug delivery

## Abstract

The present investigation aimed to formulate and optimize sustained release proliposome dry powder for inhalation of Voriconazole (VZ) and its in vitro and in vivo evaluation. The proliposome-based dry powder for inhalation was formulated by spray drying technique using Phospholipon 90H and cholesterol in the lipid phase, mannitol as a carrier, and L-leucine as a dispersing agent. A face-centered central composite design was used to study the influence of factors on responses, vesicle size, VZ entrapment efficiency, and drug release. The optimized formulation was further characterized by FTIR, FESEM, DSC, XRD, and evaluated for in vitro drug release, in vitro aerosol deposition, and in vivo lung retention study in Wistar rats. For the optimized batch F-5 proliposome formulation, vesicle size was observed as 191.7 ± 0.049 nm with PDI 0.328 ± 0.009, entrapment efficiency as 72.94 ± 0.56%, and cumulative drug release after 8 h of dissolution was 82.0 ± 0.14%. The median mass aerodynamic diameter (MMAD) generated by optimized formulation F5 was significantly lower (3.85 ± 0.15 µm, *p* < 0.0001) as compared to spray-dried voriconazole (SD-VZ) (8.35 ± 0.23 µm). In vivo studies demonstrated a profound enhancement in lung retention (3.8-fold) compared to SD-VZ and oral VZ dispersion. Conclusively, proliposome formulation of voriconazole is a plausible and convincing approach for pulmonary fungal infections, considering its sustained release behaviour and prolonged lung retention.

## 1. Introduction

Among the various fungal infections, pulmonary aspergillosis is noticeable in immune-compromised, critically ill patients, and it is characterized by cavitary lesions with paracavitary infiltrates or subsequent cavity development [1,2]. Pulmonary aspergillosis impacts around 3 million individuals globally, with Asia bearing a disproportionately high impact compared to other regions [3,4]. The recent statistics state the global incidence of pulmonary aspergillosis, which corresponds to 50–90% of cases of fatal pulmonary aspergillosis, prevailing with chronic obstructive pulmonary disease (COPD), tuberculosis, sarcoidosis [5], compromised immune systems, chemotherapy, and stem cell or organ transplantation. Estimates for the year 2019 indicates that 1.2 million individuals had chronic pulmonary aspergillosis (CPA) from pulmonary tuberculosis, over 410,000 from allergic broncho-pulmonary aspergillosis, and over 72,000 from pulmonary sarcoidosis [6,7,8]. Pulmonary aspergillosis is also observed in hospitalized individuals with severe influenza who receive large doses of corticosteroids [5,9]. As per the medication therapy, the aspergillus species are effective against three classes of anti-fungal agents from the existing five. These include echinocandins (capofungin, micafungin), triazoles (itraconazole, voriconazole, posaconazole), and polyenes (amphotericin B).

Voriconazole (VZ) is the first-line medication for pulmonary aspergillosis [10,11]. Derived by a structural modification of fluconazole, VZ is an FDA-approved BCS class II antifungal drug characterized by its low solubility and high permeability [12]. It has a poor water solubility (0.098 mg/mL), pKa of 2.01 and 12.7, and log P of 1.82 [13]. VZ is known to suppress Cytochrome P450 (CYP 450)-dependent 14-lanosterol demethylation, which is an essential step in synthesizing ergosterol in fungal cell walls [14,15]. The inhibition of the production of ergosterol further leads to the lysis of the fungal cell wall [16]. The maximal plasma concentration of VZ occurs within 1 to 2 h after oral treatment, attaining approximately 96% of bioavailability. However, clinical investigations show a wide range of absorption (35–83%) associated with high or low CYP2C19 enzyme activity [17,18,19]. The variation in VZ concentration influences cytochrome P450-dependent metabolism of other drugs, resulting in drug interactions and clinical problems such as organ abnormalities and toxicity. VZ is available in both oral and parenteral forms and leads to a variety of adverse consequences such as photopsia, skin rashes, elevated levels of liver enzyme, and abdominal discomfort [20]. Oral voriconazole administration may have a distinct hepatotoxicity profile from IV dosing due to high portal circulation concentrations triggering liver enzyme abnormalities [21,22,23]. There is a substantial possibility of failure in obtaining therapeutic efficacy when the drug is administered via oral or IV routes. The reasons are inefficient drug concentration at the site of action due to higher elimination rate and reduced blood flow because of the angio-invasive hyphae of the fungus, possibilities of higher systemic side effects, and occurrence of resistance [24,25]. The adverse effects associated with the systemic availability of VZ have emphasized the need for alternative methods of drug delivery systems [26]. Considering above drawbacks, pulmonary drug delivery system (PDDS) seems promising to resolve the abovementioned issues and provide a site-specific treatment of VZ in pulmonary aspergillosis [27]. A drug dose in situ will remain undiluted by the overall circulation, reducing systemic toxicity and enhancing therapeutic efficacy at the required site [25]. Several findings related to VZ administration through the pulmonary route have been reported in the literature. The disposition of VZ has also been studied after inhalation in rodents and humans. The research on dry powder inhalation therapy of voriconazole formulated as highly respirable powders [28], controlled release non-porous and porous nanoparticles [29,30], controlled release polylactide microparticles [31], and amorphous or crystalline VZ insufflations using thin-film freezing method [32] has been reported with their improved in vitro and in vivo performance via pulmonary route. As stated in the previous studies, an increase in solubilization of VZ would increase its serum availability and lung distribution [33,34].

Considering its low, and pH dependent, aqueous solubility, VZ can be incorporated into the lipid-based system in order to improve solubility and lung tissue availability [35]. Formulation of liposomes is one of the most preferred drug delivery approaches for poorly soluble antifungal drugs. Liposomes are small spherical single or multi-layered vesicles made of phospholipids and cholesterol with an aqueous phase in their core. Liposomes are suitable for incorporating both hydrophilic and lipophilic drugs and exhibit site-specific action and prolonged retention time [36]. A study on lipid vesicular based aerosol system of voriconazole demonstrated marked improvement in drug permeability through the lung epithelial barrier and significant retention in the lung following nebulization [37]. Despite being one of the most effective drug targeting approaches, liposome formulation has a few disadvantages regarding its physicochemical stability on storage and the complexity involved in the preparation methods, like thin film hydration related to their scale up. These drawbacks limit the use of liposomal products on a large commercial scale [38]. These complications can be catered to by formulating free-flowing dry particles/powder called proliposomes, which form a liposomal dispersion upon hydration with the medium or biological fluid [39]. Proliposomes are lipid vesicles that enclose the drug of interest within their bilayer structure. They are completely dry, freely flowing powders [40]. Proliposomes have superior stability characteristics compared to other lipid solutions/suspensions since they are obtained and kept dry. Proliposomes’ dry powder form is effective for transport and storage, reducing expenses that would otherwise be required. To become liposomes, proliposomes undergo a quick and uncomplicated hydration phase beneath temperature and agitation control. Proliposomes function locally or, if required, immediately absorb and enter the systemic circulation after being quickly hydrated in the aqueous environment of the lungs [41].

The spray drying technique has been optimized and employed by various formulation scientists to formulate dry powder for inhalation of the desired particle size (<5 µm) which is essential for deep lung penetration. The preparation of dry powders for inhalation can be scaled up efficiently using spray drying, as it is a single-step, non-tedious process that allows for the optimization of multiple parameters, including solvent type, solid content, and instrumental conditions, to control moisture content, size distribution, particle size, and morphology (i.e., temperature, solution feed rate, gas supply and use of different types of nozzles) [42,43]. Various studies have demonstrated applications of spray drying methods in preparation of stable proliposome based dry powder inhalation formulations and correlation of their in vitro and in vivo characteristics with therapeutic efficacy [44,45,46,47]. In contrast to conventional pulmonary drug delivery systems, dry powder inhalations (DPI) provide a viable approach for administering drugs to lower airways and deep lung tissues [48]. Furthermore, the stability of the drug is maintained during prolonged storage periods due to the dry nature of DPI [41]. Dry powder inhalers (DPIs) are susceptible to microbial contamination because they are administered directly to the respiratory system, necessitating stringent sterilization protocols to ensure patient safety [49]. Reference studies emphasized the critical importance of proper cleaning and disinfection procedures for inhaler devices, including DPIs, in reducing the risk of infections caused by microbial colonization and focus on their qualitative analysis. Effective sterilization methods, such as gamma irradiation, ethylene oxide sterilization, and dry heat sterilization, are required to eliminate microorganisms and preserve product integrity [50].

In the present study, a proliposome-based dry powder inhalation formulation of VZ was developed. This system has not previously been reported in the literature for pulmonary targeting of voriconazole. The proliposome DPI was prepared by spray drying technique and optimized using DOE (Design of experiment) approach. The Design of Experiments (DOE) approach is useful in optimizing Voriconazole proliposome formulations because it systematically evaluates various factors and their impact on product performance. DOE can also characterize interactions between formulation parameters, allowing the development of robust proliposome formulations with improved drug encapsulation efficiency and stability. In the present study, the factors were first screened by fractional factorial design as screening design to identify the critical ones. The selected parameters were further investigated using a face-centered central composite design. The formulation was assessed for VZ entrapment, vesicle size upon dilution, powder flow properties, morphology by scanning electron microscopy, characterization by FTIR, DSC, XRD, in vitro drug release, and aerosol performance by cascade impactor and in vivo lung retention studies.

## 2. Materials and Methods

Voriconazole (VZ) was procured from Dr. Reddy’s Laboratories, Hyderabad, India, as a gift sample. Cholesterol, L-leucine, and D-mannitol were purchased from Hi-Media Mumbai. Phospholipon 90H was procured as a gift sample from Lipoid, Ludwigshafen, Germany. Organic solvents and other materials were of analytical or HPLC grade.

### 2.1. Production of VZ Proliposome Formulation (VZF) Based DPI by Spray Drying Method

Single-step spray drying process was utilized in the formulation of VZ proliposome-based DPI. Briefly, the lipid phase (500 mg) comprising Phospholipon (PH) and cholesterol (CH), and pure voriconazole drug (PD-VZ) were added to ethanol (30 mL) to form the solution. L-leucine (LC) (10% *w*/*v*) as a dispersing agent and D-mannitol (MN) as a carrier was added to water (70 mL) to obtain the aqueous solution. The lipid solution was added to the aqueous phase with constant stirring with an overhead stirrer for 30 min. A translucent mixture was obtained that was further sonicated for 15 min before spray drying. The above mixture was then subjected to spray drying (Labultima-LU222, Mumbai, India) using the following parameters: inlet and outlet temperatures as 120 ± 5 °C and 70 ± 5 °C, respectively, with a spray rate of 2–4 mL/min. The proliposome powder was collected from cyclone chambers in a tightly closed container and stored in desiccators until use [45,46,47]. To compare the performance of proliposome formulation with spray-dried drug, pure drug voriconazole (PD-VZ) alone was spray-dried (SD-VZ) using the same parameters as optimized proliposome formulation.

### 2.2. Screening Design

The process and formulation parameters affecting proliposome spray-dried powder formulation were identified. A 2^4−1^ fractional factorial design was utilized to screen the critical parameters from variables, lipid:drug mass ratio (A), ratio of Phospholipon 90H:Cholesterol mass ratio in the lipid phase (B), amount of mannitol as a carrier (C), and spray rate (D). The critical variables were identified based on their effect on responses, vesicle size (Y_1_), and VZ entrapment efficiency (Y_2_). This screening design (2^4−1^) enabled the study of the impact of four different parameters with only eight experiments (Table 1). A multiple linear regression analysis was employed to estimate the main effects of factors [51]. The critical parameters were identified based on the ANOVA results and Pareto chart.

### 2.3. Optimization Design

In accordance with the findings of the screening design, the effect of critical variables was further investigated by employing a face-centered central composite design. The design comprises two factors at three levels, each with an alpha (α) value specified as one. The effect of factors, Lipid:Drug mass ratio (X1) at levels −1 (1:1), 0 (2:1), +1 (3:1) and amount of mannitol (X2) at levels −1 (500 mg), 0 (1500 mg), +1 (2500 mg) on vesicle size of liposome (Y1) and drug entrapment (Y2) in liposome was investigated. Thirteen runs were conducted (Table 2) and data underwent analysis employing Design Expert (Stat-Ease, version 10, Minneapolis, MN, USA) software. Multiple linear regressions were conducted to confirm the main and interactive effects. The influence of variables on the responses was also assessed using contour and 3D surface plots.

### 2.4. Characterization of DPI Formulation

Proliposome powder (20 mg), of concentration 1 mg/mL, was dispersed by sonication for 10 min in phosphate buffer saline (PBS), pH 7.4 (20 mL). The resultant liposomes were assessed for their vesicle size and VZ entrapment in order to investigate the impact of formulation and process parameters on responses.

#### 2.4.1. Vesicle Size

Vesicle size was examined by dynamic light scattering technique employing a particle size analyzer (Horiba, SZ-100, Osaka, Japan). The size measurement was performed at a fixed 90° angle and a temperature of 25 °C. The Z-average diameter and polydispersity index (PDI) were obtained.

#### 2.4.2. Percent Entrapment Efficiency (%EE)

The liposome dispersion (5 mL) was introduced in a dialysis bag (Himedia, Molecular size cut off 12,000 to 14,000 Dalton) and the two ends of the bag were tied [52]. This bag was further immersed in a 50 mL PBS (pH 7.4) medium with stirring at 100 rpm, and the temperature was maintained up to 37 ± 5 °C. After 60 min, the free drug in the media was evaluated using a UV Spectrophotometer (Shimadzu, UV-1900, Kyoto, Japan) at maximum absorption wavelength (λmax) of 256 nm (Molecular formula of VZ C_16_H_14_F_3_N_5_O). The drug entrapment efficiency (% EE) was calculated as follows. The experiment was conducted in triplicate, and the mean of the measurements was utilized [53,54].

$$\%\;\mathrm{EE}=\frac{Total\;Drug-Unentraped\left(free\right)\;Drug}{Total\;Drug}\times 100$$

#### 2.4.3. Micromeritic Properties

##### The Angle of Repose

The material was carefully poured through the funnel, previously adjusted to a fixed height (i.e., 2 cm). The powder was poured until it reached the point where the formed pile touched the tip of the funnel. Accordingly, radii were obtained, and the angle calculation was performed [55].

##### Bulk Density and Tapped Density

The powder was poured into a measuring cylinder to determine the bulk density. The same cylinder was further tapped about 500, 750, or 1000 times until there were no further changes in the bulk volume of the powder. Carr’s compressibility index and Hausner’s ratio were determined based on the bulk density [56].

#### 2.4.4. Morphology by Field Emission Scanning Electron Microscopy (FESEM)

The surface morphology of SD-VZ and optimized proliposome formulation VZF (F5) was assessed using FESEM (Nova nano SEM, 450, Lincoln, NE, USA). Before the assessment, samples were coated with a thin metal layer and secured on a sample mount. The samples were scanned under a vacuum in the FESEM chamber. Photomicrographs were captured at different magnifications at an acceleration voltage of 15–18 kV to examine morphological differences.

#### 2.4.5. Fourier-Transform Infrared Red Spectroscopy (FTIR)

Infrared absorption spectra of PD-VZ, PH, LC, CH, MN, PM, SD-VZ, and VZF were recorded using FTIR Spectrophotometer with ATR (Brucker Alpha-T FTIR) across a 600–4000 cm^−1^ range.

#### 2.4.6. Differential Scanning Calorimetry (DSC) Study

To validate the formation of proliposomes, a differential scanning calorimetry study of PD-VZ, excipients such as PH, LC, MN, CH, PM, SD-VZ, and VZF was conducted using PerkinElmer DSC system (4000, Shelton, CT, USA). Each sample weighing 2 mg was transferred into a sealed aluminium pan and exposed to heating in the range of 30 to 350 °C, with a nitrogen flow rate of 20 mL/min and a heating rate of 10 °C/min. Temperature calibration was done using an empty aluminium pan as a reference.

#### 2.4.7. X-ray Diffraction (XRD) Analysis

XRD analysis (Rigaku, Miniflex 600, Tokyo, Japan) of PD-VZ, PH, LC, MN, CH, PM, SD-VZ, and VZF was conducted to determine the crystallinity changes that occurred during the formation of proliposomes by using Cu Kα rays at a current of 15 mA and a voltage of 40 kV. The scanning was performed at a diffraction angle 2θ in the 20 to 80° range to obtain a diffraction pattern.

#### 2.4.8. In Vitro Drug Release

The drug release of the DPI formulation was assessed in vitro using a modified USP dissolution test equipment type I with dialysis membrane. The dialysis membrane ensures separation of released drug molecules from proliposomes, allowing them to easily pass through into the release medium. This method also eliminates the need to separate the released compounds from proliposomes, making sampling relatively simple and eliminating unwanted loss during sample preparation and handling [57]. For the study, the dialysis membrane of molecular size cut off 12,000 to 14,000 Dalton (Himedia) was soaked overnight in the dissolution medium before the experiment. Proliposome powder (equivalent to 5 mg of PD-VZ) was dispersed in 5 mL of PBS (pH 7.4) and sonicated for 10 min. The dispersion was introduced into the dialysis tube. The tube was tied from both ends and further hitched to the stirrer shaft. The dissolution was performed in PBS pH 7.4 (100 mL) at 37 ± 5 °C. The dissolution sample was withdrawn at one-hour intervals up to 8 h, diluted appropriately, and drug release in the media was examined using a UV spectrophotometer (Shimadzu, UV-1800, Kyoto, Japan) at 256 nm. At sampling intervals, aliquots of medium were replenished in order to maintain the sink conditions. To elucidate the drug release mechanism from the proliposome formulations, release kinetic models including zero order, first order, Higuchi matrix, and Korsmeyer–Peppas were applied (PCP Disso V3, India). The correlation coefficient values and release rate constant were obtained to confirm the model fitting [58].

#### 2.4.9. Laser Diffraction Study of Powder

The aerodynamic particle size distribution of spray dried proliposome powder was assessed by laser diffraction analyzer using dry sampler attachment (Horiba, LA960, Osaka, Japan) (Refractive index during measurement: 1.6 and air pressure: 0.3 mPa). The particle size was expressed as d_10_ (representing 10% of particle in the sample with given size or less), d_50_ (representing 50% of particle in the sample with given size or less), and d_90_ (representing 90% of particle in the sample with given size or less) and mean.

#### 2.4.10. In Vitro Aerosol Performance

The aerodynamic particle size distribution of SD-VZ and optimized VZF was determined by Anderson Cascade Impactor (ACI) (Copley, UK) with a separator. The experiment was carried out by applying a vacuum to the ACI under the flow rate of 60 L/min for 10 sec and the air pressure maintained was p3/p2 ≤ 0.5 to ensure steady state air flow and pressure drop (P1) across inhaler was maintained to 4.0 Kpa. Every stage plate was pre-coated with glycerol to avoid the bounce back of the particles upon aerosolization. The powder to be evaluated was covered in a “size 3” hard gelatine capsule (Health Caps India Ltd., Sahibzada Ajit Singh Nagar, India) and aerosolized using Lupihaler (Lupin Ltd., Mumbai, India). The capsules (five) loaded with SD-VZ and VZF powder equivalent to 10 mg of the PD-VZ, were taken for each test (impaction), respectively. The test was performed in triplicate. A solubilizing solvent, methanol, was employed to disperse the sample retained in the capsule and inhaler device, induction port, pre-separator, plates of all stages (with aerodynamic cutoff diameter of 9 μm, 5.8 μm, 4.7 μm, 3.3 μm, 2.1 μm, 1.1 μm, 0.7 μm, 0.4 μm for stages S0 to S7), and filter of the ACI. The drug content was then evaluated using an Agilent 1120 Compact binary gradient Liquid chromatography system with UV detector. Kromacil C-18 (250 × 4.6 mm, 5 μm) column was used as the stationary phase, and the mobile phase was 0.1 M Acetate Buffer (pH 3.0): acetonitrile (40:60) with 1 mL/min flow rate and examined at 256 nm. The concentrations obtained were utilized to calculate the aerosolized parameters of the product, i.e., mass median aerodynamic diameter (MMAD), fine particle fraction (FPF), and emitted dose (ED) [59].

#### 2.4.11. In Vitro Antifungal Activity

In order to ensure the antifungal efficacy of the drug and its formulation, in vitro agar diffusion assay was used [60,61,62]. For this study, the agar plates were inoculated with a standardized culture of *Candida albicans* (1 × 10^8^ cfu/mL) using sterilized sabouraud dextrose agar as a medium. *C. albicans* overnight cultures (0.1 mL each) were evenly distributed on the corresponding agar media poured to a depth of 4 mm and bored three 9 mm diameter wells for the samples, Voriconazole proliposome formulation (VZF), pure Voriconazole drug (PD-VZ), and solvent control. In order to ensure the complete release of encapsulated voriconazole drug, VZ proliposomes (200 mg) were dispersed in DMSO (10 mL) and Triton X-100 (100 μL), followed by incubation at room temperature for 1 h. Addition of Triton X-100 ensured disruption of liposome membrane. The dispersion was centrifuged at 3000 rpm to obtain the supernatant containing the released voriconazole. The released voriconazole from the proliposomes, pure Voriconazole in DMSO, and Triton X-100 in DMSO as control are each added to their respective wells. The plates were placed in the refrigerator for 30 min to allow diffusion and incubated for 48 h at 37 ± 2 °C under aerobic conditions to promote fungal growth. The diameters of the inhibition zones that form around each well were measured [61].

#### 2.4.12. In Vivo Lung Retention Study

The in vivo lung retention or lung tissue availability of SD-VZ and VZF was compared with that of oral dispersion (OD-VZ) in Wister rats. For this study, Wister rats of either sex were taken and fasted overnight before the study. The study was conducted with strict adherence to the guidelines of the committee to control and supervise experiments on animals (CPCSEA), Ministry of Environment and Forest, Government of India and the protocol for the same was approved by institutional animal ethics committee. The rats were provided with food ad libitum [63] and housed individually at 25 °C under a 12-h light-dark cycle. The animals were grouped into four groups, with each containing 18 animals. Each group was divided into subgroups of 3 animals per time point. Group 1 was control with no treatment provided. Group 2 animals were administered with Voriconazole oral suspension (OD-VZ) (10 mg/kg). Group 3 and Group 4 animals were directly insufflated SD-VZ (10 mg/kg) and VZF (10 mg/kg) respectively. The DPI formulation was administered by direct insufflations in the tracheal region using a powder insufflator designed for rodents in our laboratory. The time points considered were 0.5, 1, 2, 4, 8, and 24 h. At each time interval, 3 animals in the sub-group were sacrificed by cervical dislocation, and lungs were harvested. The excised lungs were dried and weighed. The lung tissues were sliced with the cutter and homogenized for 10 min with pre-cooled dichloromethane in an ice bath. The mixture was centrifuged at 10,000 rpm (Refrigerator centrifuge, Remi, ZFCU-07420, Mumbai, India) for 10 min. The supernatant was collected and to 0.5 mL of it, 0.1 mL of Amlodipine (50 µg/mL) was added as internal standard. The supernatant was evaporated to dryness, reconstituted with 1 mL of mobile phase, mixed by vortexing, and centrifuged. The supernatant was filtered through a syringe filter (0.22 µm) and reverse-phase HPLC analysis was performed on the filtrate. The mobile phase set was 30 mM acetate buffer (pH 3.0): Acetonitrile (50:50). The flow rate was 1 mL/min, and detection was done at 256 nm. Analysis was conducted using an Agilent 1120 Compact LC binary gradient system with UV detector and Kromacil C-18 (4.6 × 250 mm, 5 μm) column was used as a stationary phase. The maximum peak concentration of the drug in tissue (Cmax) and the area under the concentration-time curve (AUC 0→24) were determined as pharmacokinetic parameters using PK solver add-in program in Microsoft Excel (Microsoft Corporation, Redmond, WA, USA). A one-way analysis of variance was used to investigate statistical variance, followed by the Bonferroni multiple comparison test (GraphPad InStat 3, San Diego, CA, USA). Statistical significance was ascribed to a *p*-value of ≤0.05.

## 3. Result and Discussion

### 3.1. Formulation and Screening of Proliposome DPI

The performance of the proliposome formulation depends on its ability to form liposomes upon rapid hydration. The vesicle size of the formed liposomes and the drug entrapment (EE) in its structure is very critical for the therapeutic effectiveness and targeting. There are various formulation and process variables that may affect the vesicle size and EE. The formulation components such as nature of lipids like phospholipids, cholesterol, their concentration with respect to the drug, ratio of phospholipid to cholesterol, and amount of carrier with respect to lipids are some of the critical parameters that will affect the liposome characteristics [64]. The process parameters with respect to spray drying including nozzle size during spraying, spray rate, inlet temperature, etc., would impact performance of DPI [65].

During preliminary optimization, the critical parameters affecting proliposome formulations were screened using (2^4−1^) fractional factorial design. The effects of Lipid:Drug ratio (A), Phospholipon 90H:Cholesterol ratio (B), amount of mannitol as a carrier (C), and spray rate for spray drying (D) on vesicle size (Y_1_) and VZ entrapment (Y_2_) upon hydration, polydispersity index, and flow property were studied. These factors were selected for screening design based on preliminary experiments. The screening data demonstrated Lipid:Drug ratio (A) and the carrier concentration (C) significantly impacted the percent entrapment efficiency and the vesicle size. The vesicle size was found in the range of 203.9 nm to 305.9 nm, whereas entrapment efficiency of eight batches were found in the range of 53.2 ± 0.5% to 92.2 ± 0.3%. The angle of repose of the formulations was in the range of 33.6 to 42.7°. The ANOVA test was performed to determine statistical significance, followed by the student *t*-test (Table 3).

**Figure 1:**
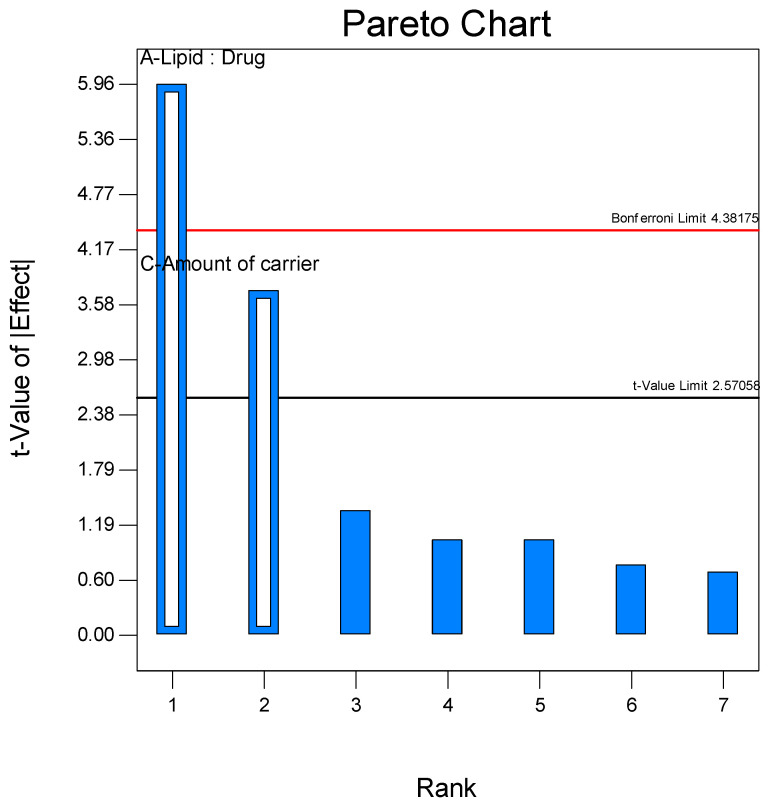
Pareto chart showing the effect of factors Lipid:Drug (A) and amount of carrier (C) on the Entrapment Efficiency.

In the half-normal plot, Lipid:Drug ratio (A) and amount of mannitol as a carrier (C) revealed substantial deviation from the straight line, indicating a significant influence of these factors on vesicle size and entrapment efficiency of proliposome formulation. However, feed rate (D) did not demonstrate any significant effect. The Pareto chart (Figure 1) further confirmed this, which presented higher t-values (above the Bonferroni limit) of factors A and C.

Phospholipon 90H:Cholesterol ratio in lipid blend (B) significantly affected vesicle size and polydispersity indices of the vesicles but did not reveal any effect on drug entrapment. Enhancing the cholesterol proportion in the lipid blend decreased the vesicle size and polydispersity index of the formulation. The addition of cholesterol imparts rigidity to the vesicle layer and also decreases gel to liquid phase transition [66]. This imparts flexibility to the vesicle bilayers during higher inlet temperature of spray drying resulting in lower vesicle size with narrow particle size distribution. As the Phospholipon 90H:Cholesterol ratio did not substantially affect the drug entrapment, it was not further considered in the optimization design. For optimizing the correct Phospholipon 90H:Cholesterol ratio, additional formulations with ratios of 1:2 and 2:1 were evaluated. Considering the size of the vesicles (213.9 nm) resulting from 1:2 ratio compared to that of 2: 1 ratio (1594.9 nm), Phospholipon 90H:Cholesterol ratio of 1:2 was fixed for further study. The amount of carrier affected the angle of repose of the formulation. The addition of mannitol to the formulation reduced the adherence of the powder to the cyclone during spray drying. The minimum amount of carrier required for good flow of DPI was found to be 1:1 (500 mg of mannitol). With further increase in mannitol amount, there was less aggregation of powder particles enhancing their flow properties.

Considering the significant effect of parameters A and C on critical quality attributes (vesicle size and drug entrapment efficiency), these two parameters were chosen for further design experiments. The insignificant factor, the spray rate, was set at a constant value of 3 mL/min. To obtain improved flow properties and dispersibility of proliposome formulations, L-leucine (10% *w*/*v*) was added as a dispersing agent. The addition of L-leucine further reduces particle aggregation during spray drying and imparts the desired aerodynamic characteristics to the powder [67,68,69].

### 3.2. Optimization Using Face-Centered Central Composite Design

The effect of Lipid:Drug ratio (X_1_) and amount of mannitol (X_2_) on proliposome formulation was further studied using a face-centered Central Composite design (CCD) [70]. The Central Composite design is a comprehensive factorial design that incorporates center points and sets of axial points, enabling the estimation of curvature in the experimental space. The performance of proliposome formulation depends on the vesicles obtained after hydration of proliposome formulation. Therefore, spray-dried proliposome formulations were hydrated to determine vesicle size (Y_1_), drug entrapment (Y_2_), and drug release (Y_3_). The factor X_1_ (Lipid:Drug mass ratio) was studied at three levels 1:1 (−1), 2:1 (0), 3:1 (+1) and variable X_2_ (amount of mannitol) was studied at levels 500 mg (−1), 1500 mg (0), and 2500 mg (+1). The vesicle size ranged from 164 ± 0.2 nm to 371 ± 0.2 nm, VZ entrapment was in the range of 54 ± 0.77% to 91 ± 0.54%, and drug release was in the range of 57 ± 0.39% to 100 ± 0.16% (Table 2). ANOVA was used to evaluate statistical significance, and then a student t-test was performed (Table 4).

The effect of Lipid:Drug ratio (X_1_) and amount of mannitol (X_2_) on vesicle size (Y_1_), VZ entrapment efficiency (Y_2_), and drug release after 8 h (Y_3_) is represented in Equations (1)–(3) as a result of multiple linear regression analysis.

Y_1_ = + 202.66 − 28.17X_1_ − 18.55X_2_ + 10.87X_1_X_2_ − 65.72X_1_^2^ + 97.13X_2_^2^ (1)

Y_2_ = + 71.20 − 4.56X_1_ − 9.71X_2_ − 1.77X_1_X_2_ − 7.35X_1_^2^ + 9.12X_2_^2^ (2)

Y_3_ = + 87.77 + 79.99X_1_ + 3.00X_2_ − 0.43X_1_X_2_ − 12.30X_1_^2^ + 7.53X_2_^2^ (3)

**Figure 2:**
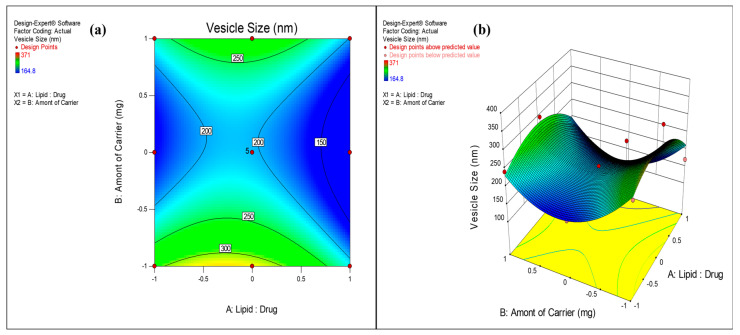
(**a**) Contour plot and (**b**) response surface graph showing the effect on vesicle size.

**Figure 3:**
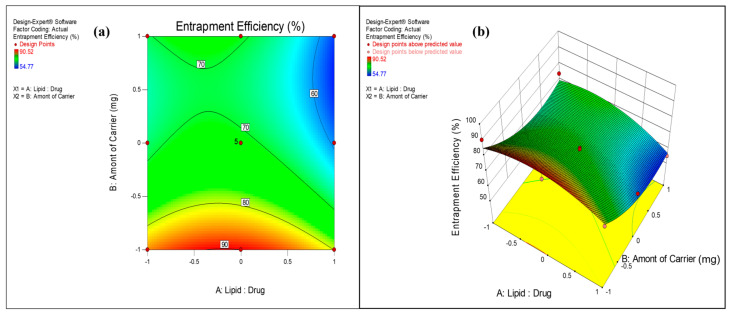
(**a**) Contour plot and (**b**) response surface graph showing the effect on Entrapment Efficiency.

**Figure 4:**
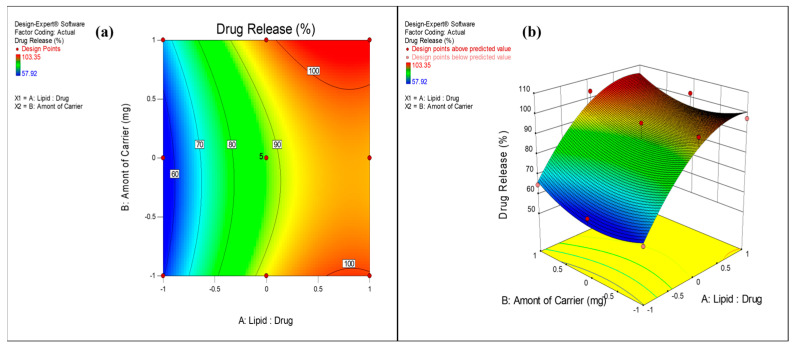
(**a**) Contour plot and (**b**) response surface graph showing the effect on drug release.

The surface response plots and contour plots (Figure 2, Figure 3 and Figure 4) were further used to depict the relationship between factors and responses.

#### 3.2.1. Effect on Vesicle Size

The performance of the proliposome powder depends on its resultant vesicle size upon hydration. Previous studies have reported that size less than 200 nm is critical for nanovesicles in order to permeate through mucosal barriers and enter into lung epithelium, circumventing the alveolar macrophages [71]. In the applied CCD, vesicle size of the formulations was obtained in the range of 164 ± 0.2 nm to 371 ± 0.2 nm. The impact of X_1_ and X_2_ on vesicle size was well described by the quadratic model. Higher F value and *p* value less than 0.05 (0.036) implied the significance of model. However, higher *p* values of X_1_ (0.1274) and X_2_ (0.2923) indicated non-significance of these model terms. This implies factors, Lipid:Drug ratio and amount of mannitol did not significantly affect the vesicle size. Presence of X_1_^2^ and X_2_^2^ in the quadratic Equation (1) indicated nonlinearity of the response. It is evident from the surface response and contour plots (Figure 2) that the lowest vesicle size was obtained at middle levels (1500 mg) of mannitol concentration. The optimum amount of carrier is required to form a uniform lipid coat around the particles which, after hydration, forms uniform vesicles. The lower amount of carrier is insufficient for uniform coating and hence causes aggregation of particles, resulting in larger vesicles. At the highest level of mannitol (2500 mg), the amount of lipid was insufficient to coat the carrier, leading to non-uniform size distribution and agglomeration. The polydispersity index of the formulations was in the range of 0.260 to 0.425, indicating broad size distributions of vesicles. Lower PDI value (closer to 0.1) demonstrates more uniformity in vesicle size distribution.

#### 3.2.2. Effect on Entrapment Efficiency

As proliposome powder is prepared by single step spray drying technique, there is a chance that drug may get adsorbed on the surface of particles or remain free in the powder instead of getting entrapped in the lipid. Therefore, assessment of drug entrapment efficiency in liposome is critical parameter in optimization of proliposome formulation. Considering the lipophilic nature of VZ, entrapment efficiency in liposome was good and found in the range of 54 ± 0.77% to 91 ± 0.54%. The Lipid:Drug ratio and amount of carrier had a substantial impact on drug entrapment. Quadratic model was the best fit model (*p* value 0.0089) to depict the effect of these variables on entrapment efficiency. The lower *p* values (0.0613 and 0.0021) indicated significant impact of X_1_ and X_2_ on drug entrapment. The effect of these variables was nonlinear as evident from the presence of squared terms in the equation. Higher ratio of signal to noise (adequate precision > 4) indicated the suitability of model to navigate the design space. It is evident from the contour plot and surface response plot (Figure 3) that the VZ entrapment was higher (>80%) at lower mannitol concentration (500 mg). An increase in mannitol, as a carrier, decreased the drug entrapment in liposomes. This could be attributed to improved solubility and partitioning of VZ in the aqueous phase by mannitol resulting in low entrapment efficiency. The drug entrapment was lower (<60%) at higher level of carrier (2500 mg) concentration and at higher Lipid:Drug mass ratio (3:1). The drug entrapment was higher at a Lipid:Drug ratio of 2:1. Increase in the Lipid:Drug ratio from 1:1 to 2:1 increased the drug entrapment. The higher amount of lipid could entrap more drugs in its vesicular structure resulting in higher entrapment. Further increase in lipid concentration decreased drug entrapment significantly.

#### 3.2.3. Effect on Drug Release

The impact of Lipid:Drug mass ratio and amount of mannitol on drug release (after 8 h value) was best explained by quadratic model and Equation (3). The positive coefficient of X_1_ and X_2_ in the equation indicated an increase in drug release with an increase in the value of Lipid:Drug ratio from 1:1 to 3:1 and the amount of mannitol from 500 mg to 2500 mg. The higher F value (14.17) and *p* value less than 0.05 (0.0014) indicated significance of the model. From ANOVA results (Table 4), it is evident that the factor, Lipid:Drug mass ratio (X_1_) exerted a substantial influence on drug release (*p* value 0.0001) whereas the amount of mannitol did not show significant effect (*p* value 0.2819). Figure 4 depicts the increase in drug release with an increase in Lipid:Drug ratio from level (−1) to level (1). VZ has a log *p* value of 1.8 with intermediate polarity. Due to this, it is entrapped in the vesicle with its orientation towards the hydrophilic head near the surface of the vesicles, resulting in higher drug release from proliposome formulation.

Considering the responses, vesicle size, drug entrapment, and drug release desirability constraints were applied on the responses for optimizing the proliposome formulation. The desirable vesicle size was less than 200 nm, drug entrapment was greater than 70% and drug release was more than 80%. Micromeritic properties of the formulation were also considered while optimizing the formulation. For all powder formulations, the angle of repose was observed in the range of 25° to 38°, Carr’s index was in the range 8 to 44%, and the Hausner ratio was observed as 1.08 to 1.8. Formulation (VZF) with 2:1 (0 level) Lipid:Drug mass ratio and 1500 mg of mannitol (0 level) was considered as optimized with vesicle size 191.7 ± 0.1 nm with PDI 0.328, entrapment efficiency as 72.9 ± 0.56%, and cumulative drug release after 8 h of dissolution was 82.0 ± 0.1%. The optimized batch demonstrated excellent micromeritic properties (angle of repose of 25°, Carr’s index of 8%, and Hausner ratio of 1.08). In order to validate the model, the batch was repeated and re-evaluated and predicted Vs experimental values for vesicle size, entrapment efficiency, and drug release were compared. The percent prediction error was 5.3% which was less than 10%, thus validating the model.

### 3.3. FESEM Analysis

**Figure 5:**
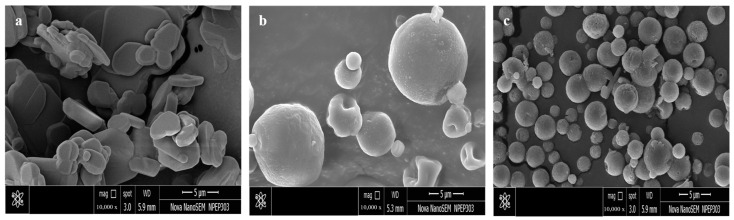
FESEM images of (**a**) Pure Voriconazole drug (PD-VZ), (**b**) Spray dried drug (SD-VZ), and (**c**) Voriconazole proliposome formulation (VZF).

The FESEM of pure drug PD-VZ indicated crystalline nature of the drug, whereas FESEM of SD-VZ and VZF showed smooth spherical shaped particles (Figure 5).

SD-VZ particles indicated larger size (>10 µm) compared to VZF (<10 µm). This was further confirmed by particle size analysis. The surface of SD-VZ had dimples or grooves whereas the surface of VZF was smooth. The smooth surface of VZF could be because of the adsorption of leucine on the surface of the particles. It is reported that the rough surface properties led to inferior flow whereas smooth surfaces result in improved flow properties. This can be correlated to higher values of the angle of repose of SD-VZ (39°). A few agglomerated particles are visible in VZF which could be due to lipid in the formulation. As the lipid is coated on the mannitol surface during spray drying, it could have resulted in agglomeration.

### 3.4. Fourier-Transform Infrared Red Spectroscopy

**Figure 6:**
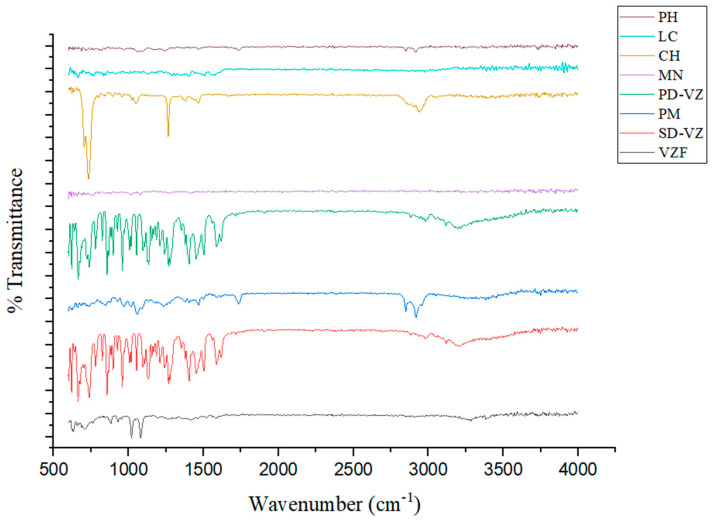
IR spectra of Phospholipon 90H (PH), L-leucine (LC), cholesterol (CH), D-mannitol (MN), voriconazole pure drug (PD-VZ), physical mixture (PM), spray dried drug (SD-VZ), and optimized proliposome formulation (VZF).

The FTIR analysis of PH, LC, CH, MN, PD-VZ, PM, SD-VZ, and VZF was performed to establish evidence of the absence of any chemical interaction of PD-VZ with other excipients employed in the formulation (Figure 6).

The FTIR spectrum of Phospholipon 90H exhibited the typical C-H symmetric and asymmetric signals for the long fatty acid chain at 2849.40 cm^−1^ and 2916.48 cm^−1^, respectively. The C=O stretching band at 1735.19 cm^−1^ indicates the presence of fatty acid esters. The spectra showed P=O stretching bands at 1242.94 cm^−1^, P-O-C stretching bands at 1172.59 cm^−1^, and −N + (CH3)_3_ stretching at 967.25 cm^−1^. The characteristic infrared spectra of L-leucine are attributed to several functional groups, including the germinal dimethyl group, amino group, and carboxyl group. Strong infrared absorption was observed in the areas including 3253.03 cm^−1^, 3410.89 cm^−1^, and 1570.64 cm^−1^ for OH and C=O, respectively. The aliphatic CH3 bending of the methyl group produced prominent infrared absorption spectra at 1402.25 cm^−1^. Carbonyl group (C=O) stretching vibrations in amide bonds were detected at 1570.64 cm^−1^ (amide I) and 1503.35 cm^−1^ (amide II). In case of cholesterol, at 1275.12 cm^−1^, a peak was identified for C-O stretching. The bands ranging from 2902.42 cm^−1^ to 2940.15 cm^−1^ in cholesterol can be attributed to the aliphatic CH group’s asymmetric and symmetric stretching vibrations, respectively. One double band (C=C) is present in the second ring of cholesterol and was prominently shown at 1630.13 cm^−1^, with C-H bending observed at 1464.48 cm^−1^, and C-O stretching at 1264.90 cm^−1^. Absorption peaks characteristic of mannitol from 3246.30 cm^−1^ to 3498.18 cm^−1^ indicated OH- stretching, whereas a peak at 2996.22 cm^−1^ indicated C-H stretching.

The FTIR spectra of VZ showed two prominent peaks, one at 2983.63 cm^−1^, which indicated the C-H stretching of the CH_3_ group, and another at 3195.29 cm^−1^, which was a wider peak reflecting the OH group. There was a noticeable aromatic stretch of the C=N bond between 1400.11 cm^−1^ and 1499.80 cm^−1^. C-H (aliphatic) was indicated by a peak at 1589.01 cm^−1^, whereas C-F stretching was shown at 1130.60 cm^−1^. Out-of-plane C-H (aromatic) bending was seen in bands at 859.96 cm^−1^ and 781.26 cm^−1^, which correspond to 1, 2, and 4 tri-substituted aromatic rings. The infrared (IR) spectra of VZ and SD-VZ are quite identical, demonstrating that spray drying had no major influence on the molecular structure. Both spectra showed nearly identical peaks, suggesting no significant changes in chemical composition. The presence of an additional band at 1615 cm^−1^ of C=C conjugated stretching in the spray-dried sample suggests minor structural modification. The broader peaks in PM spectra ranged from 3331.61 cm^−1^ to 3383.11 cm^−1^, indicating OH groups. A distinctive band at 1588.79 cm^−1^ was identified as a C=C aromatic stretch, confirming the existence of PD-VZ. A band at 1232.52 cm^−1^ indicated C=N stretch. The FTIR spectrum of VZF exhibits characteristic peaks that indicate the voriconazole presence in formulation such as the C-H and C-F stretching vibrations at 2437.84 cm^−1^ and 1081.59 cm^−1^, respectively. Furthermore, the voriconazole is further confirmed by the C=N group at 1273.57 cm^−1^ and the -C=C stretch at 1583.53 cm^−1^. The presence of out-of-plane C-H (aromatic) bending at 879.09 cm^−1^ and 709.34 cm^−1^ further supports the presence of the trisubstituted aromatic ring characteristic of voriconazole. The presence of significant peaks corresponding to the drug indicates that the drug and other proliposome formulation components interact minimally, confirming the integrity and stability of VZ in VZF.

### 3.5. DSC Analysis

**Figure 7:**
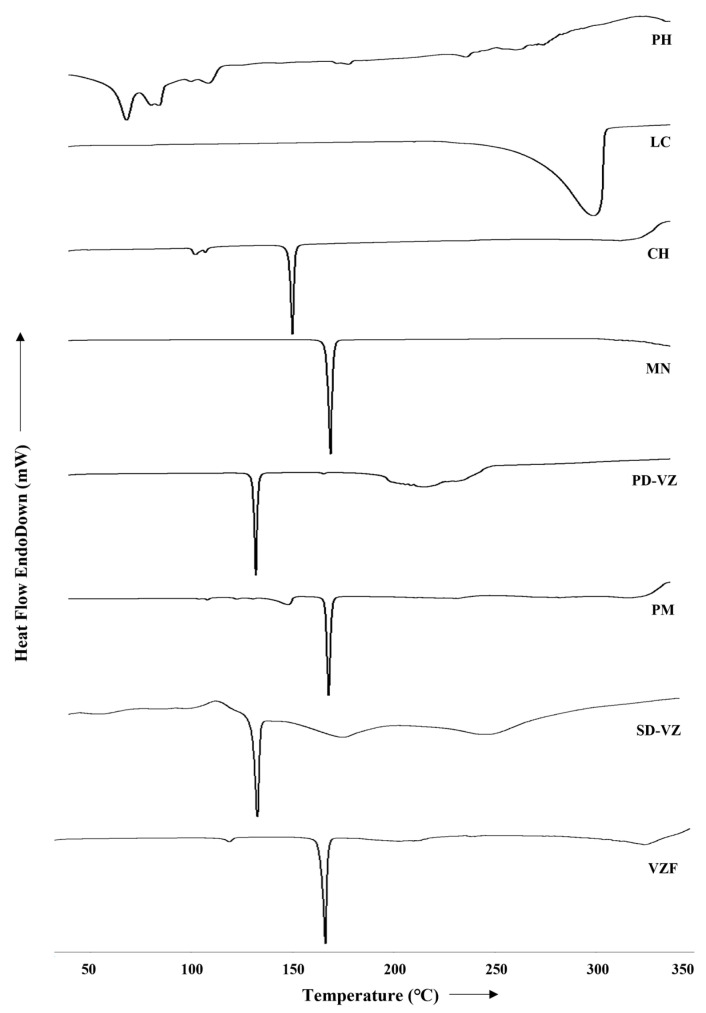
DSC Thermograms of Phospholipon 90H (PH), L-leucine (LC), cholesterol (CH), D-mannitol (MN), voriconazole pure drug (PD-VZ), physical mixture (PM), spray dried drug (SD-VZ), and optimized proliposome formulation (VZF).

The DSC thermograms were analyzed to evaluate the physical transformation of the drug from crystalline to amorphous state (Figure 7). DSC thermograms of MN, LC, CH, PH, PD-VZ, PM, SD-VZ, and VZF were compared. At 130.97 °C, PD-VZ demonstrated a sharp endothermic peak that indicated its crystalline nature and melting point. The DSC thermogram of MN appeared to have a prominent sharp endothermic peak at 168.59 °C, indicating its melting point. CH and PH revealed many sharp and broad peaks, possibly because lipids are composed of different components. A sharp peak of CH appeared at 149.3 °C. A broad peak is seen in the DSC of leucine at 304.51 °C. No sharp endothermic peak of VZ was identified in the formulation or physical mixture. The physical mixture indicated peaks at 147.41 °C, 167.6 °C, and above 300 °C, representing CH, MN, and LC. As these excipients were in excess in the mixture compared to other ingredients, the VZ and PH peaks were not visible. SD-VZ retained the sharp melting endotherm of VZ at 130.9℃, indicating its crystalline nature. As VZ is practically insoluble in water, during spray drying the drug was dispersed in solvent and retained its crystalline nature even after spray drying. In the proliposome formulation, a small peak at 118.92 °C indicated VZ, a sharp peak at 166.46 °C represented MN, and a peak near 300 °C confirmed the presence of LC. A very small and broad peak of VZ endotherm in VZF indicated its conversion into an amorphous state during spray drying.

### 3.6. XRD Analysis

**Figure 8:**
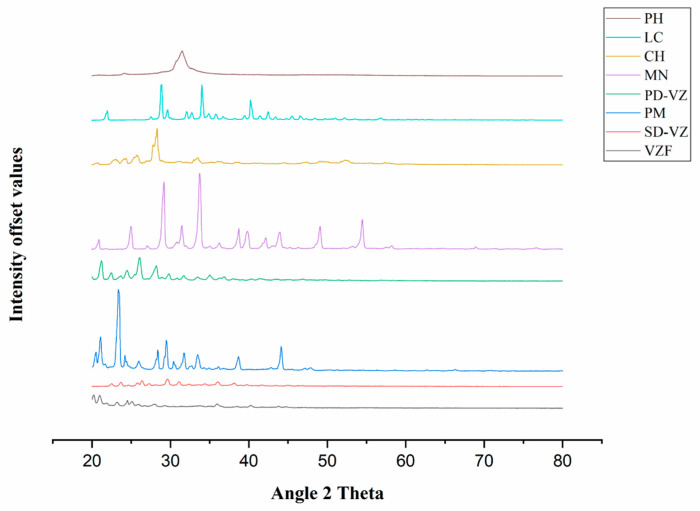
X-ray Diffractogram of Phospholipon 90H (PH), L-leucine (LC), cholesterol (CH), D-mannitol (MN), voriconazole pure drug (PD-VZ), physical mixture (PM), spray dried drug (SD-VZ), and optimized proliposome formulation (VZF).

X-ray diffractogram of the MN, LC, CH, PH, PD-VZ, PM, SD-VZ, and VZF (Figure 8) were evaluated to study the changes in the drug crystallinity during proliposome formulation. PD-VZ diffractogram demonstrated high-intensity peaks at 21.2°, 22.46°, 23.7°, 24.46°, 26.06°, 28.18°, and 29.76° that are characteristic of VZ. CH and PH showed characteristic diffraction patterns in the range of 22.48° to 23.46° and 23.98° to 24.32°, respectively. LC and MN demonstrated crystalline diffraction patterns with characteristic peaks for LC from 28.74° to 28.94° and for MN at 20.88°, 24.98°, 29.18°, 31.46°, 33.74°, and 38.72°. In the PM, few distinct peaks were observed at 20.58°, 21.08°, 23.46°, 24.2°, 28.38°, 29.46°, 33.46°, 38.68°, and 44.12°. This represented a large quantity of excipients present in the mixture, mostly MN. The VZ characteristic peaks were also detected in the physical mixture. The X-ray diffractogram of SD-VZ and formulation VZF indicated a decline in the intensity of the characteristic peaks of VZ and other ingredients observed in the diffractogram of the physical mixture. This demonstrated a change in the crystallinity of the drug during spray drying. A few sharp peaks at the characteristic 2Ɵ position of VZ indicated the presence of the drug in partial crystalline (semi-crystalline) form. In proliposome formulation, a few sharp peaks in the range of 20° to 21° indicated the presence of mannitol also in a semi-crystalline state. These observations agree with the DSC results.

### 3.7. Drug Release and Drug Release Kinetics

**Figure 9:**
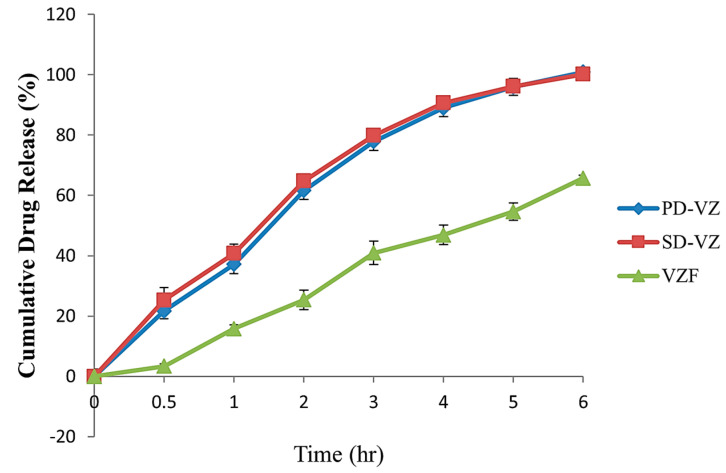
Comparison between pure drug (PD-VZ), spray-dried drug (SD-VZ), and optimized proliposome formulation (VZF).

The drug release of optimized proliposome formulation VZF was compared with spray dried drug, SD-VZ, and pure drug, PD-VZ, and results are presented in Figure 9. In vitro drug release studies indicated 100% of the drug release in 6 h of dissolution from SD-VZ and PD-VZ. The drug release after 30 min from PD-VZ and SD-VZ was found to be 21.7 ± 0.5% and 25.3 ± 0.7% respectively. The drug release from optimized VZF batch was 3.3 ± 0.6% in 30 min, 65.7 ± 0.2% in 6 h, and 82.1 ± 0.2% in 8 h. This demonstrated sustained release of VZ from proliposome formulation compared to SD-VZ and PD-VZ. There was no difference in the drug release of spray dried and pure drug Voriconazole. This could be due to similar particle size distribution. As voriconazole is a BCS class II drug with poor aqueous solubility, 6 h were needed for complete release of the drug through the dialysis membrane. In liposomes, orientation of VZ (log P 1.8) is towards the hydrophilic head of the phospholipid layer. During dialysis, the drug molecules first partitioned from liposome bilayer to donor fluid in the bag and then further partition into receptor or dissolution media. This could have sustained the initial (30 min) as well as overall release of VZ.

The data on drug release was subjected to various release kinetic models (Table 5) to deduce the drug release mechanism. The regression coefficient values and *n* value of Korsemeyer–Peppas (KP) was considered to confirm the model fitting [72,73]. Zero order model describes the drug release as independent of concentration of drug in reservoir or dosage form. Thus, the release of drug is constant with time irrespective of amount of remaining drug in the system. In first order model, the drug release from the system depends on the remaining amount of drug in a drug delivery system resulting in continuous change in drug release rate. Higuchi matrix model emphasizes diffusion as the major drug release mechanism whereas Korsemeyer–Peppas model is used when drug release is by non-fickian mechanism, or more than one drug release mechanism is involved. The *n* value of Korsemeyer–Peppas equation describes the mechanism of drug release. If *n* value is 0.5, then KP equation changes into simplified Higuchi equation explaining diffusion through polymer matrix as the drug release mechanism. When value of *n* varies between 0.5 to 0.9, the drug release is by non-fickian mechanism. The drug release in this case is due to multiple mechanisms including diffusion across a matrix and erosion of the polymer or lipid matrix. If *n* value of K-P equation is 0.9 or 1.0, then drug release follows zero order kinetics and the release rate is constant [74,75]. Considering the *n* value of VZF (1.03), the drug release mechanism was verified to follow zero-order kinetics. Thus, the proliposome formulation ensured constant drug release, whereas the drug release mechanism for SD-VZ was non-fickian or anomalous. In liposome formulation, the lipid bilayer slowly deteriorates with time releasing the drug in the dialysis bag. From the dialysis bag, the drug is then released into receptor fluid at a constant rate. The higher regression coefficient of Hixson Crowell model for VZF formulation (0.9953), further confirms this theory. The Hixson Crowell kinetics are followed by the system which loses their mass during dissolution. The deterioration of lipid bilayer could be one of the reasons for the mass reduction over time.

### 3.8. Laser Diffraction Study of Powder

The preferred size distribution of inhaled particles for targeting to various areas of the respiratory tract is well defined. For deep lung targeting, the particle size of formulation should be restricted in the range of 0.5 to 7 µm. The particle size of DPI is determined in terms of aerodynamic diameter by various methods including cascade impactor and laser diffraction. Cascade impactors are considered as a standard technique recommended by pharmacopoeia owing to its relevance to particle deposition in the respiratory tract. Currently, laser diffraction is also a preferred technique for size measurements of DPI. Various comparative studies have been reported for particle size measurement by these two techniques and, although a correlation in particle size can be established with these two techniques, deviation in cumulative size fractions is also reported [76,77]. As VZ proliposome is expected to enter into the deeper lung region, to ensure the smaller size distribution of proliposome powder, the size was determined by laser diffraction and in vitro aerosol performance of sample was also observed.

**Figure 10:**
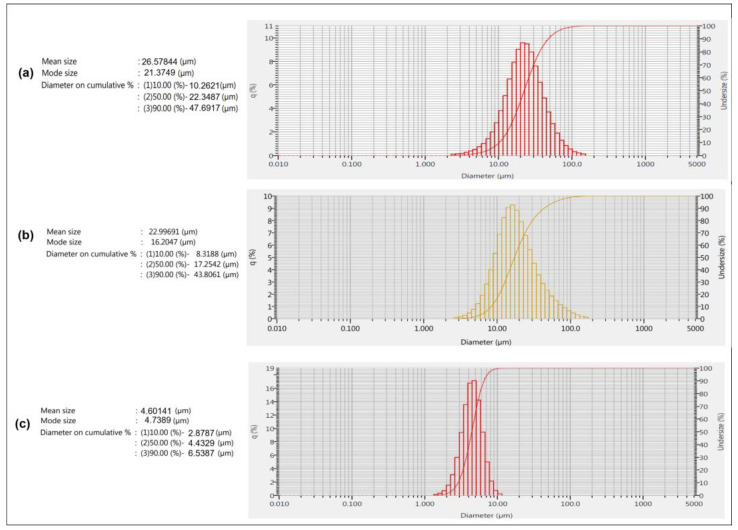
Laser diffractogram of (**a**) Pure Voriconazole drug (PD-VZ), (**b**) Spray dried drug (SD-VZ), and (**c**) Voriconazole proliposome formulation (VZF).

Figure 10 indicates particle size distribution of pure drug (PD-VZ), spray dried drug (SD-VZ), and proliposome formulation (VZF). The mean size of PD-VZ was 26.5 µm, and D50 was 22.3 µm, indicating 50% of the sample with size below the indicated size. D90 of the PD-VZ was 47.6 µm indicating 90% of sample below this size. The mean size of SD-VZ was 22.9 µm, D50 and D90 was 17.2 µm and 43.8 µm respectively. For VZF formulation, mean size was 4.6 µm, D50 and D90 were 4.4 µm and 6.5 µm respectively. Thus, the size of VZF formulation is found in the desired range as recommended for DPI. The excipients like mannitol and L-leucine in VZF are responsible for reduction in agglomeration of particles by imparting hydrophilicity to powder. Leucine is also known to disperse and aerosolize the DPI particles and improves their aerodynamic size. To confirm these results further, in vitro aerosol performance of the proliposome DPI was determined.

### 3.9. In Vitro Aerosol Performance

**Figure 11:**
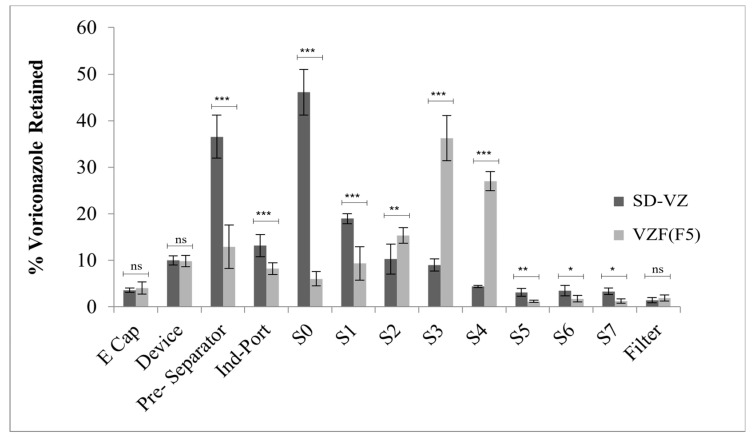
Drug deposition on various stages of cascade impactor for spray dried voriconazole (SD-VZ) and voriconazole proliposome formulation (VZF) batch F5. The error bar represents the standard deviation. Not significant (ns) indicates *p* > 0.05, * indicates *p* < 0.05, ** indicates *p* < 0.01 and *** indicates *p* < 0.001.

The fraction of drug deposited on various stages of Anderson Cascade Impactor (ACI) [78] for VZF (F-5) and SD-VZ was compared (Figure 11).

The median mass aerodynamic diameter (MMAD) generated by VZF powder was significantly lower (3.85 µm, *p* < 0.0001) as compared to SD-VZ (8.35 µm) [71,72]. In SD-VZ, a substantial amount of powder was retained in the pre-separator, whereas, for VZF formulation, a considerable powder fraction was retained in stages 3 and 4, having a cut-off diameter of 3.3 µm and 4.7 µm, respectively (Table 6). The percent emitted dose (ED) for VZF and SD-VZ was 85–90%. Fine particle fraction (FPF) value (<4.7 µm) showed a correlation to MMAD values and was significantly higher in VZF compared to SD-VZ (Table 7). MMAD of VZF correlates to that of aerodynamic size obtained by laser diffraction (4.6 µm) whereas there was significant difference in MMAD of SD-VZ by cascade (8.35 µm) and size determined by laser (22.9 µm).

Use of mannitol and Leucine improved the fine fraction in the formulation, whereas in SD-VZ, these two excipients were not used. Leucine acts as a dispersing agent that acts on the attractive forces between the particles and reduces the size of the atomized droplet during spray drying due to its surfactant properties. In order for the DPI to transit the lower respiratory tract, its particle size must be within the range of 1–7 µm. The particles larger than 10 µm may get retained in the upper respiratory tract and rapidly eliminated by the lung’s clearance mechanism; the particles smaller than 1 µm are easily removed from the lungs by diffusion. The lower MMAD (1–7 µm) and higher fine particle fraction of VZF indicated compliance with this requirement. Rojanarat et al. [46] highlighted the role of mannitol in MMAD of proliposome formulation. The non-porous mannitol allows the lipid layer to coat on its surface during spray drying. However, particles are agglomerated due to the external phase of lipids. Using microporous mannitol could further reduce the size of the powder as lipids will be deposited in porous structures rather than the surface.

### 3.10. Antifungal Activity

**Figure 12:**
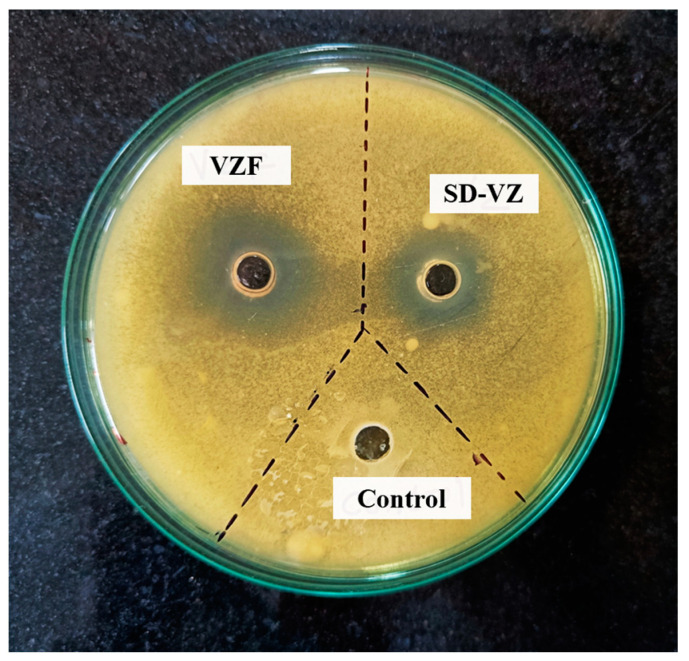
Antifungal activity of VZF, VZ and positive control (Triton X-100) against *C. albicans* after 24 h of incubation. The figure showcases the potential growth inhibition of *C. albicans* by VZF, providing its potential as an antifungal formulation.

The antifungal activity of Voriconazole proliposome formulation (VZF), the pure Voriconazole drug (VZ), and Triton X-100 in water as a solvent control was performed by using agar well diffusion assay (Figure 12). Triton X-100 was used in the assay to enable the drug release from liposome formulation. The drug release from liposome occurs in vivo due to lipolysis by endogenous lipase or the lipase secreted by aspergillus or fungal species at the site of infection. From Figure 12, it is evident that the VZF had a substantially larger zone of inhibition (ZOI) at 18 mm than the PD-VZ with 11 mm ZOI (Figure 10). The combination of carriers in the formulation improved drug solubility, stability, and overall effectiveness, contributing to the increased antifungal activity. The solvent control, Triton X-100 in water, demonstrated no inhibition, confirming its role as a non-antifungal agent. This emphasizes the specificity of the observed antifungal effects in Voriconazole-containing samples. Thus, the study indicates that the Voriconazole proliposome formulation enriched with carriers inhibits fungal growth more effectively than both the pure drug and the control.

### 3.11. In Vivo Lung Retention Study

The in vivo lung retention of optimized VZF was compared with SD-VZ and OD-VZ in Wistar rats. The drug in the lung extract was analyzed using a previously developed HPLC method using a UV detector at 256 nm. The retention time for PD-VZ and amlodipine as internal standards was observed at 5.7 and 4.2 min, respectively. The linearity was observed in the 0.5 to 10 μg/mL range.

**Figure 13:**
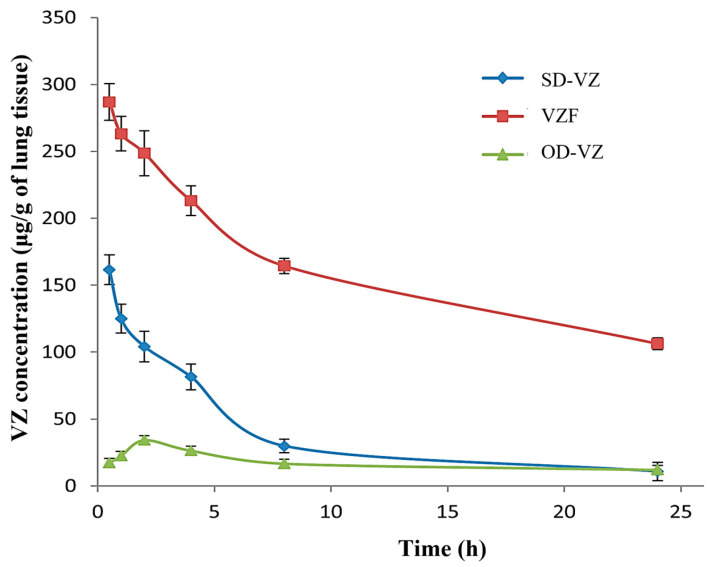
In vivo lung retention of Voriconazole formulations; spray dried voriconazole (SD-VZ), voriconazole proliposome formulation (VZF), Voriconazole oral dispersion (OD-VZ).

Figure 13 indicates the lung retention profile of SD-VZ and VZF by inhalation route and PD-VZ dispersion by oral route. The pharmacokinetic parameters for lung retention are shown in Table 8.

The lung uptake of PD-VZ (AUC_0–24h_) was found to be 417.81 μg·g^−1^·h, 1010.89 μg·g^−1^·h, 3924.84 μg·g^−1^·h respectively when administered as oral dispersion, SD-VZ inhalation, and VZF proliposome inhalation. The tissue availability of VZ was found to be significantly (*p* < 0.001) increased in VZF. The enhancement in the lung distribution was approximately 3.8-fold compared to spray-dried voriconazole inhalation. For effective management of infections, the anti-infective drug should be retained in the target area for a longer time. VZF formulation demonstrated sustained release behavior, and the VZ was retained in the lung for a prolonged period in VZF formulation compared to oral dispersion and SD-VZ powder. MMAD is a critical parameter in DPI which plays a significant role by influencing the deposition and subsequent clearance of aerosolized particles in lungs. The size distribution determined by MMAD affects particle residence time in the respiratory tract, thereby influencing clearance mechanisms, which is critical for understanding drug efficacy in pulmonary drug delivery. MMAD less than 5 to 7 μm is essential for deposition and retention of particles in lower respiratory tract. Considering the lower MMAD of VZF (3.85 μm) than SD-VZ (8.35 μm), the lung retention of VZ in VZF was significant. The conventional spray-dried formulation (SD-VZ) had a higher MMAD due to its retention primarily in the upper respiratory tract, resulting in lower lung penetration and voriconazole concentration. Initial lung concentrations of both the formulations can further confirm this. The initial concentration of SD-VZ (161.45 μg·g^−1^) was lower than the VZF formulation (286.95 μg·g^−1^). This contrast illustrates the superior efficacy of VZF proliposomes in achieving targeted pulmonary delivery and emphasizes the critical role of MMAD in determining drug deposition patterns.

## 4. Conclusions

In the current investigation, we have successfully formulated a proliposome-based dry powder for inhalation of voriconazole using a simple and single-step spray drying strategy. This formulation was developed using screening design before optimization. The particle size of the developed formulation was determined to be within a range (<5 µm) of respirable particles required for deep lung penetration in the pulmonary region. The in vitro aerosol performance employing a cascade impactor demonstrated drug deposition of FPF in the lower region of the lungs. In vivo studies of the voriconazole proliposome formulation demonstrated a sustained release and enhanced retention time compared to spray-dried voriconazole inhalation and oral formulation. Therefore, the voriconazole proliposome formulation developed was found to be an optimistic approach for delivering voriconazole to the lungs for the treatment of fungal infection due to its substantial, sustained release and prolonged retention time at the site of action.

## Figures and Tables

**Table 1:** Screening of factors using fractional factorial Design.

RUN	Factors			
	Lipid:Drug (A)	Phospholipon 90H:Cholesterol (B)	Amount of Carrier (mg) (C)	Spray Rate (mL/min) (D)
S-1	1 (4:1)	−1 (1:1)	−1 (500)	1 (4)
S-2	−1 (1:1)	−1 (1:1)	1 (2500)	1 (4)
S-3	−1 (1:1)	−1 (1:1)	−1 (500)	−1 (2)
S-4	−1 (1:1)	1 (1:4)	−1 (500)	1 (4)
S-5	1 (4:1)	−1 (1:1)	1 (2500)	−1 (2)
S-6	−1 (1:1)	1 (1:4)	1 (2500)	−1 (2)
S-7	1 (4:1)	1 (1:4)	1 (2500)	1 (4)
S-8	1 (4:1)	1 (1:4)	−1 (500)	−1 (2)

For each formulation, the quantity of lipid was fixed as 500 mg, and the drug amount was changed as per the level. The factors and their levels in the Table 1 are; Lipid:Drug mass ratio (A) at levels −1 (1:1), +1 (4:1); and Phospholipon 90H:Cholesterol mass ratio (B) at levels −1 (1:1), +1 (1:4); Amount of mannitol as carrier (C) at levels −1 (500 mg), +1 (2500 mg) and spray rate during spray drying (D) at −1 (2 mL), +1 (4 mL).

**Table 2:** Experimental design with actual levels of variables and responses.

BATCH	X_1_ (Lipid:Drug Mass Ratio)	X_2_ (Amount of Carrier in mg)	Vesicle Size (nm) * Y_1_	PDI *	Entrapment Efficiency (%) * Y_2_	Drug Release in 8 h (%) * Y_3_
F-1	3:1(+1)	1500	174.7	0.377	62.87 ± 0.3	101.2 ± 0.9
F-2	2:1(0)	1500	177.9	0.400	72.48 ± 0.7	95.3 ± 0.7
F-3	2:1(0)	500	371.0	0.288	86.42 ± 0.2	97.1
F-4	2:1(0)	2500	294.2	0.300	68.43 ± 1.0	101.3 ± 0.2
F-5	2:1(0)	1500	191.7	0.328	72.94 ± 0.6	82.0 ± 0.1
F-6	3:1(+1)	2500	175.2	0.339	54.77 ± 0.5	100.2 ± 0.8
F-7	1:1(−1)	1500	164.8	0.348	59.05 ± 0.2	58.8 ± 1.0
F-8	1:1(−1)	2500	242.9	0.267	74.71 ± 0.3	64.7 ± 0.6
F-9	2:1(0)	1500	204.7	0.376	71.91 ± 0.2	82.6 ± 0.9
F-10	3:1(+1)	500	170.7	0.363	78.46 ± 0.6	97.1 ± 0.4
F-11	1:1(−1)	500	281.9	0.421	90.52 ± 0.3	57.9 ± 0.1
F-12	2:1(0)	1500	191.4	0.330 ± 0.97	72.44	86.6 ± 0.2
F-13	2:1(0)	1500	182	0.352	72.0 ± 0.2	82.3 ± 0.8

* Values are expressed as Mean ± SD (*n* = 3).

**Table 3:** Data of ANOVA for responses for screening design.

Source	Sum of Squares		Mean of Square		F Value		*p*-Value (Prob > F)	
	Y_1_	Y_2_	Y_1_	Y_2_	Y_1_	Y_2_	Y_1_	Y_2_
Model	2.408 × 10^5^	1088.38	1.204 × 10^5^	544.19	6.43	24.72	0.0415	0.0026
A	-	782.10	-	782.10	-	35.53	-	0.0019
B	1.195 × 10^5^	-	1.195 × 10^5^	-	6.38	-	0.0528	-
C	1.213 × 10^5^	306.28	1.213 × 10^5^	306.28	6.47	13.91	0.0516	0.0136

**Table 4:** Data of ANOVA of responses for central composite design.

Source	F Value			*p*-Value (Prob > F)		
	Y_1_	Y_2_	Y_3_	Y_1_	Y_2_	Y_3_
Model	4.52	7.79	14.57	0.0367	0.0089	0.0014
X_1_- Lipid:Drug	2.99	4.96	60.31	0.1274	0.0613	0.0001
X_2_- Amt. of Carrier	1.30	22.47	1.36	0.2923	0.0021	0.2819
X_1_X_2_	0.30	0.50	0.019	0.6026	0.5035	0.8948
X_1_^2^	7.49	5.91	10.50	0.0290	0.0453	0.0142
X_2_^2^	16.37	9.11	3.94	0.0049	0.0194	0.0876

**Table 5:** Release Kinetics of spray-dried formulations.

Code	Zero-Order	First Order	Matrix		Korsemeyer–Peppas		Hixson Crowell
	R^2^	R^2^	R^2^	K	R^2^	*n*	R^2^
SD-VZ	0.9290	0.9339	0.9941	2.0478	0.9965	0.6368	0.9323
PD-VZ	0.9403	0.9446	0.9921	1.9771	0.9961	0.6683	0.9432
VZF	0.9949	0.9855	0.9507	1.2597	0.9931	1.0372	0.9953

**Table 6:** Data Interpretation for in vitro aerosol performance.

Stage	Cut-Off Diameter (μm)	Drug Retained (mg)		Fraction Retained		% Fraction Retained		% Cumulative (More than)		% Cumulative (Less than)	
		SD-VZ	VZF (F5)	SD-VZ	VZF (F5)	SD-VZ	VZF (F5)	SD-VZ	VZF (F5)	SD-VZ	VZF (F5)
E Cap	-	1.73	1.99	0.03	0.04	3.56	4.08	-	-	-	-
Device	-	4.85	4.81	0.01	0.10	10.00	9.85	-	-	-	-
Pre-Separator	-	17.72	6.32	0.36	0.12	36.56	12.94	-	-	-	-
Ind-Port	-	6.38	4.01	0.13	0.08	13.15	8.21	-	-	-	-
S0	9	8.21	1.93	0.47	0.06	46.10	6.04	46.10	6.04	53.91	93.97
S1	5.8	3.37	2.99	0.19	0.09	18.94	9.36	65.04	15.40	34.96	84.61
S2	4.7	1.82	4.87	0.10	0.15	10.25	15.33	75.29	30.73	24.71	69.28
S3	3.3	1.61	11.47	0.09	0.36	9.01	36.24	84.30	66.97	15.70	33.04
S4	2.1	0.78	8.54	0.04	0.26	4.40	26.99	88.69	93.96	11.31	6.05
S5	1.1	0.56	0.38	0.03	0.01	3.13	1.18	91.82	95.15	8.18	4.86
S6	0.7	0.61	0.55	0.03	0.02	3.47	1.73	95.30	96.88	4.70	3.13
S7	0.4	0.59	0.40	0.03	0.01	3.31	1.25	98.61	98.12	1.39	1.89
Filter	-	0.25	0.61	0.01	0.02	1.41	1.89	100.02	100.02		

**Table 7:** Parameters for in vitro aerosol performance.

Parameter	SD-VZ	VZF (F5)
Mass Median Aerodynamic Diameter (MMAD) (μm)	8.35 ± 0.23	3.85 ± 0.15
Fine Particle Fraction (FPF) (<4.7 μm)	12.82 ± 0.18	54.86 ± 0.05
% Emitted Dose (ED)	86.43 ± 0.13	86.07 ± 0.13

Values are expressed as Mean ± SD (*n* = 3).

**Table 8:** Pharmacokinetic parameters for lung retention study.

Lung Pharmacokinetic Parameters	Oral Dispersion (OD-VZ)	SD-VZ Inhalation	VZF Inhalation
Cmax (μg·g^−1^)	34.20 ± 22.96	161.45 ± 11.19	286.95 ± 5.67
AUC_0–24h_ (μg·g^−1^·h)	417.81	1010.898	3924.845
AUC_0–inf h_ (μg·g^−1^·h)	698.38	1107.723	6702.645

Values are expressed as Mean ± SEM (*n* = 3).

## Data Availability

The data is not publicly available at this time as it will be used in other ongoing studies.
